# Preferential Metabolic Improvement by Intermittent Fasting in People with Elevated Baseline Red Cell Distribution Width: A Secondary Analysis of the WONDERFUL Randomized Controlled Trial

**DOI:** 10.3390/nu13124407

**Published:** 2021-12-09

**Authors:** Benjamin D. Horne, Joseph B. Muhlestein, Heidi T. May, Viet T. Le, Tami L. Bair, Sterling T. Bennett, Kirk U. Knowlton, Jeffrey L. Anderson

**Affiliations:** 1 Intermountain Medical Center Heart Institute, Salt Lake City, UT 84107, USA; jbrent.muhlestein@imail.org (J.B.M.); heidi.may@imail.org (H.T.M.); viet.le@imail.org (V.T.L.); tami.bair@imail.org (T.L.B.); kirk.knowlton@imail.org (K.U.K.); jeffreyl.anderson@imail.org (J.L.A.); 2 Division of Cardiovascular Medicine, Department of Medicine, Stanford University, Stanford, CA 94305, USA; 3 Cardiology Division, Department of Internal Medicine, University of Utah, Salt Lake City, UT 84132, USA; 4 Department of Physician Assistant Studies, College of Medical and Health Professional Science, Rocky Mountain University of Health Professions, Provo, UT 84606, USA; 5 Intermountain Central Laboratory, Intermountain Medical Center, Salt Lake City, UT 84107, USA; sterling.bennett@imail2.org; 6 Department of Pathology, University of Utah, Salt Lake City, UT 84132, USA; 7 Division of Cardiovascular Medicine, Department of Medicine, University of California San Diego, San Diego, CA 92093, USA

**Keywords:** intermittent fasting, therapeutic fasting, RDW, RDW-CV, RDW-SD

## Abstract

Red cell distribution width (RDW) predicts cardiovascular outcomes, but it is unstudied with regard to intermittent fasting. In WONDERFUL trial subjects, the effect of the interaction between baseline RDW and intermittent fasting on changes in insulin and other cardiometabolic endpoints and the effect of fasting on changes in RDW were evaluated. The subjects enrolled were aged 21–70 years and were free of statins, anti-diabetes medications, and chronic diseases, and had ≥1 metabolic syndrome feature, as well as elevated low-density lipoprotein cholesterol. Subjects were randomized to 24-h, water-only fasting (twice per week for 4 weeks, once per week for 22 weeks) or 26 weeks of ad libitum eating. Subjects (N = 71; *n* = 38 intermittent fasting, *n* = 33 controls) had more substantial changes in insulin in intermittent fasting vs. controls (−3.45 ± 2.27 vs. 0.48 ± 3.55 mIU/L) when baseline RDW size distribution (RDW-SD) was ≥median (42.6 fL) than <median (−1.99 ± 2.80 vs. −1.08 ± 3.40 mIU/L) (*p*-interaction = 0.039). Results were similar but weaker for glucose, HOMA-IR, and metabolic syndrome score. RDW-SD (intermittent fasting: 1.27 ± 9.6 fL vs. control: −0.37 ± 1.76 fL, *p* = 0.34) was unchanged by fasting at 26 weeks. Intermittent fasting decreased insulin more in subjects with higher baseline RDW. RDW may identify individuals who derive the most health benefits from intermittent fasting and who have the most cause to adhere to a fasting regimen.

## 1. Introduction

Intermittent fasting is a popular dietary health intervention that results in weight loss of a similar level to caloric restriction, leads to improvement in some cardiometabolic risk factors, and is associated with a lower risk of chronic diseases such as coronary heart disease, heart failure, and type 2 diabetes [1,2,3,4,5,6,7,8,9,10,11]. Intermittent fasting may, however, produce a spectrum of individual health responses and even potential harms for different people depending on their health status and medical history. For example, in some populations, intermittent fasting reduced low-density lipoprotein cholesterol (LDL-C), similarly to caloric restriction [2,3], while, in others, the effect of fasting on LDL-C was not different compared to parallel controls [1,9]. Further, some people, including those with diagnosed and undiagnosed diseases, may be at risk of serious side effects from fasting, such as the risk of hypoglycemia in people with type II diabetes who are taking certain medications [12], despite the fact that intermittent fasting produces metabolic benefits for such patients [3]. The heterogeneity of benefits and risks of intermittent fasting suggests that investigations are needed to identify people who will benefit the most from fasting, thus empowering improved personalization of this dietary therapy.

Red blood cell distribution width (RDW) is a commonly obtained clinical measure of the dispersion of red blood cell (RBC) sizes (usually using the coefficient of variation (CV)). In about 1980, RDW was added to the complete blood count (CBC) laboratory panel for distinguishing between types of anemia. In 2007, the RDW-CV was reported to predict mortality in cardiology patients [13] and is now an established predictor of mortality and major adverse health events in many populations across a wide variety of diagnoses [14,15,16,17,18]. This includes its usefulness in predicting death in people diagnosed with coronavirus disease-19 (COVID-19) [19] and in apparently healthy, community-dwelling adults [20]. Other measures of the dispersion of red blood cell size exist, including the RDW size distribution (RDW-SD) that may be more predictive of mortality than RDW-CV [17].

Previously, the RDW-CV was found to be unchanged by 24-h, water-only fasting in a study of acute changes to biomarkers during one 24-h fasting period [21]. No study has evaluated the effects of repeated episodes of intermittent fasting on changes in either RDW-SD or RDW-CV, or the implications of these indicators of general health status on changes in other biomarkers in people who engage in intermittent fasting. The objectives of this study were to evaluate the change scores of RDW-SD and RDW-CV over a 26-week (6-month) period in subjects enrolled in the Weekly One-Day water-only Fasting interventional (WONDERFUL) randomized trial for intermittent fasting compared to the ad libitum control group and to test whether baseline RDW-SD or RDW-CV modified the effects of intermittent fasting.

## 2. Materials and Methods

### 2.1. Design

This study was approved by the Intermountain Healthcare Institutional Review Board (1050163) and the trial was registered at clinicaltrials.gov (NCT02770313) prior to any subject enrollment. The design and primary results of the WONDERFUL trial were previously published [9]. Briefly, subjects were randomized using a sequential block design to either intermittent fasting or an ad libitum control arm. The 24-h, water-only intermittent fasting regimen began with 4 weeks of twice-per-week fasting on non-consecutive days, followed by 22 weeks of once-per-week fasting. Intervention arm subjects ate ad libitum on non-fasting days. Subjects randomized to the control arm ate ad libitum according to their usual eating practices for 26 weeks in parallel to the intervention arm. All subjects underwent physical exams and laboratory testing, reviewed fasting logs and any nonadherence, and completed questionnaires at baseline, 4 weeks, 13 weeks, and 26 weeks.

Inclusion and exclusion criteria were also previously published [9]. Inclusions consisted of enrolling males and non-pregnant females who were aged 21–70 years, were not taking a statin, were not taking any anti-diabetes medication, had one or more criteria for the metabolic syndrome (elevated glucose, waist circumference, systolic blood pressure (SBP), diastolic blood pressure (DBP), triglycerides, or low HDL-C) and had modestly elevated LDL-C ≥ 90 mg/dL that did not reach levels that would provide a clinical indication for a statin (or, if LDL-C did meet clinical guidelines, the subject had a contraindication to statins or had previously tried them and stopped due to intolerance). Exclusions included a prior diagnosis of a chronic disease (including coronary heart disease, cerebrovascular disease, peripheral vascular disease, chronic kidney disease, chronic obstructive pulmonary disease, type 1 diabetes, dementia, immunodeficiency, and cancer), recent substantial history of deliberate fasting other than fasting for religious reasons, and (for females) being pregnant or lactating. If female participants were of child-bearing potential, they were required to commit to using study-defined contraception methods.

### 2.2. Baseline Study Variables and Outcomes

While biospecimens (plasma, DNA, RNA, stool) were collected and stored for future testing, this study utilized only clinical laboratory test results and physical exam findings from each subject visit. Clinical testing included the CBC panel (white blood cell (WBC) count, RBC count, hemoglobin, hematocrit, mean corpuscular volume (MCV), mean corpuscular hemoglobin (MCH), mean corpuscular hemoglobin concentration (MCHC), RDW-SD, RDW-CV, platelet count, and mean platelet volume (MPV)), insulin, glucose, and a lipid panel (total cholesterol, LDL-C, high-density lipoprotein cholesterol (HDL-C), and triglycerides). Each of these data elements was measured at baseline and at each follow-up study visit.

Previously reported major findings of the trial included that the low-frequency intermittent fasting regimen decreased the homeostatic model assessment of insulin resistance (HOMA-IR), a metabolic syndrome score (MSS), and insulin, with modest, but only statistically suggestive, declines in glucose [9]. Changes in those four biomarkers (insulin, glucose, HOMA-IR, and MSS) were examined as outcomes in this study, with insulin designated as the primary outcome of interest because it was most profoundly changed by intermittent fasting in the initial study results [9]. Changes in weight were also examined in this study. Each of these parameters were measured at baseline, 4 weeks, 13 weeks, and 26 weeks.

### 2.3. Statistical Considerations

Baseline characteristics of trial participants were examined overall and when stratified by RDW-SD. Evaluation of the baseline data by randomized group assignment was conducted using Student’s T-test or the chi-square test, as appropriate. Association analyses comparing the intermittent fasting and control groups for changes in RDW-SD, RDW-CV, insulin, HOMA-IR, glucose, and MSS used the intent-to-treat approach, and statistical tests were performed with Student’s T-test. The normality of distributions was evaluated with the Kolmogorov–Smirnov test prior to analyses. Tests of statistical interaction were conducted using analysis of variance to examine the modification of the effect of intermittent fasting on the study outcomes by baseline RDW. The RDW-SD was used in the primary evaluation of effect modification due to evidence that it is more predictive of clinical outcomes than the RDW-CV [17]. Correlation coefficients were calculated using Spearman’s correlation and were evaluated in the overall population. A *p*-value of *p* ≤ 0.05 was considered significant, and analyses utilized SPSS v26.0 (IBM SPSS, Armonk, NY, USA).

## 3. Results

The median baseline RDW-SD was 42.6 fL (mean: intermittent fasting, 42.9 ± 3.1 fL; controls, 42.8 ± 2.1 fL), which median was used to stratify baseline characteristics (Table 1) and to evaluate interactions with intermittent fasting. In the WONDERFUL trial [9], N = 71 subjects completed the 6-month follow-up and had CBC data to be included in this study, with *n* = 18 fasting and *n* = 17 controls having RDW-SD < 42.6 fL and *n* = 20 fasting and *n* = 16 controls having RDW-SD ≥ 42.6 fL. The proportion of subjects with higher and lower RDW-SD was similar between randomization groups (Table 1), while subjects with higher RDW-SD were older and had higher MCV, higher MPV, and lower MCHC, and tended to have greater weight and higher SBP and DBP.

Baseline RDW-SD and RDW-CV were highly correlated (*r* = 0.645), and the average RDW-CV was considerably elevated (*p* < 0.001) in the higher RDW-SD group (Table 1). Baseline RDW-SD was also modestly correlated with age (*r* = 0.384) and baseline levels of RBC count (*r* = −0.313), MCV (*r* = 0.405), MCHC (*r* = −0.366), and MPV (*r* = 0.259), but not with baseline insulin (*r* = 0.036), glucose (*r* = 0.089), HOMA-IR (*r* = 0.034), MSS (*r* = 0.16), or weight (r = 0.14). The RDW-SD change score was strongly correlated with the 26-week RDW-CV change score (*r* = 0.753) but not correlated with 26-week changes in insulin (*r* = 0.183), glucose (*r* = 0.023), HOMA-IR (*r* = 0.111), MSS (*r* = 0.169), or weight (*r* = −0.044).

**Figure 1:**
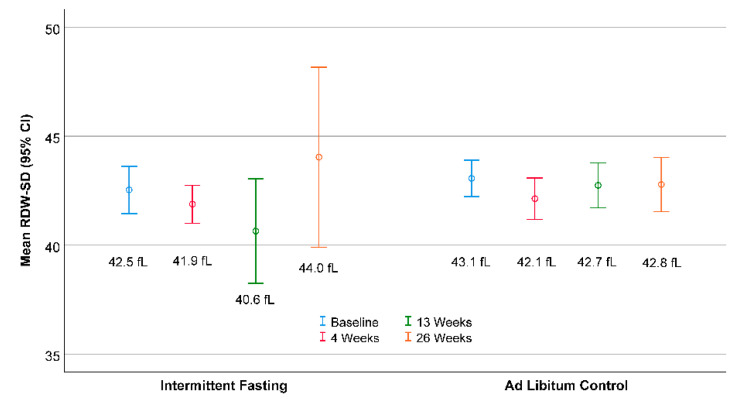
Mean (open circles, value below) RDW-SD with 95% confidence intervals (CI) (whiskers). No changes were revealed in RDW-SD for intermittent fasting compared to controls.

Mean RDW-SD levels at each timepoint are shown in Figure 1 stratified by trial randomization. No difference was found in the RDW-SD change score (the change from baseline to 26 weeks) between intermittent fasting (1.27 ± 9.61) and controls (−0.37 ± 1.76, *p* = 0.34). An outlying value was identified in one subject in which the RDW-SD rose 58.0 fL compared to baseline, and, when analysis was performed without that individual, the RDW-SD change score remained not different between intermittent fasting (−0.27 ± 1.79) and controls (−0.37 ± 1.76, *p* = 0.81).

**Figure 2:**
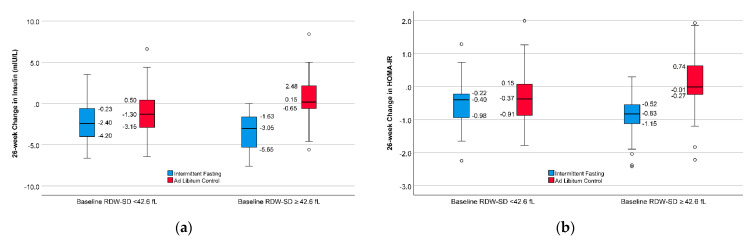

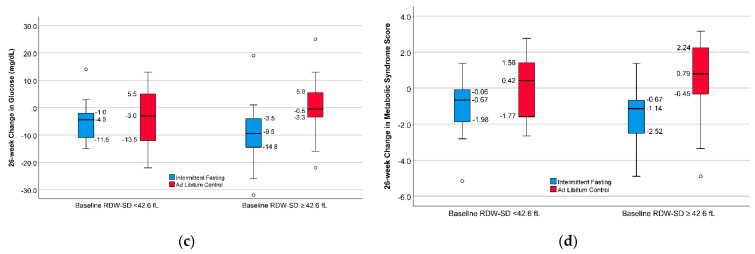
Boxplots demonstrating the 26-week change in: (**a**) insulin, (**b**) HOMA-IR, (**c**) glucose, and (**d**) metabolic syndrome score of the subjects. Change in subjects in the intermittent fasting group was greater versus controls when baseline RDW-SD was higher (≥median of 42.6 fL) than lower (<median) (bold middle lines are the median insulin, bottom and top of the boxes are 25th and 75th percentiles, and open circles are outlying values 1.5–2.9 times the height of the corresponding box).

Figure 2a shows the interaction of baseline RDW-SD with intermittent fasting in modifying the trial intervention’s effect on 26-week insulin change scores. In subjects with lower baseline RDW-SD, the change in insulin was only modest in the intermittent fasting group compared to controls (Table 2), while the decline in insulin due to intermittent fasting was profound in subjects with higher RDW-SD (Table 2), with a significant *p*-value for the interaction (*p*-interaction = 0.039). Similar findings for the effect modification by RDW-SD level on fasting were identified for 26-week change scores of HOMA-IR, glucose, and MSS (Figure 2), although these differences were not as substantial as for insulin. Mean changes in HOMA-IR, glucose, and MSS by RDW-SD and intermittent fasting categorizations are shown in Table 2.

No interaction of baseline RDW-SD and intermittent fasting was found for 26-week changes in weight (*p*-interaction = 0.33), with weight changes in the lower RDW-SD category (<42.6 fL) of −2.0 ± 5.0 kg for intermittent fasting and 0.8 ± 1.7 kg for controls, and similar weight changes in subjects with higher RDW-SD (≥42.6 fL), with −1.4 ± 4.5 kg for fasting and −0.5 ± 4.6 kg for controls. When the interaction was modified to examine the interaction of baseline weight (median: 103 kg) with intermittent fasting, no effect modification by baseline weight was found for changes in insulin (*p*-interaction = 0.13), glucose (*p*-interaction = 0.30), HOMA-IR (*p*-interaction = 0.14), or MSS (*p*-interaction = 0.29).

**Figure 3:**
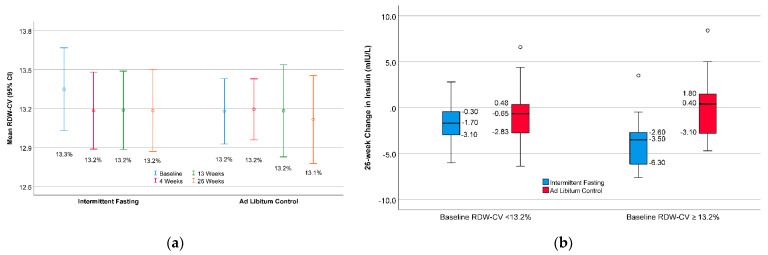
As a secondary analysis, (**a**) the mean RDW-CV (open circles) with 95% CI (whiskers) show no changes in RDW-CV for intermittent fasting versus controls. (**b**) Boxplots show that the 26-week change in insulin due to intermittent fasting (versus controls) was more substantial in subjects with higher baseline RDW-CV (≥median of 13.2%) than those with lower (<median) baseline RDW-CV.

No difference was found in the 26-week changes of RDW-CV in intermittent fasting (−0.15 ± 0.55) versus controls (−0.11 ± 0.45, *p* = 0.76) (see Figure 3a). The interaction of baseline RDW-CV (low RDW-CV: <13.2%, high RDW-CV: ≥13.2%) with insulin (Figure 3b) had *p*-interaction = 0.046, showing a similar effect modification by baseline RDW-CV as was found by baseline RDW-SD.

## 4. Discussion

### 4.1. Summary of Findings

In subjects enrolled in a randomized controlled trial of intermittent fasting, cardiometabolic outcomes, including changes to insulin level and related factors, showed improvement to a greater extent by fasting among people with a higher baseline RDW than those with an RDW of less than the median. This effect modification was the case for both RDW-SD and RDW-CV. No changes in RDW measures, however, were observed over the 6-month study interventional period. No effect modification of RDW on intermittent fasting-related weight changes was found, and no interaction of intermittent fasting with baseline weight was observed.

### 4.2. Medical Context of Elevated RDW

The RDW-CV was first reported to be a biomarker of poor health outcomes in an evaluation of the ability to predict mortality of all CBC parameters [13]. Because of its connection to anemia and calculation from basic, well-established hematologic factors, RDW-CV is abundantly available through clinical laboratory testing throughout the world. The RDW-SD is also available on some hematology platforms today and provides similar or better ability to predict health outcomes [17]. While both RDW measures are expected to be elevated in patients with certain anemias and hemorrhagic conditions, RDW-CV is also persistently elevated in people with renal failure, chronic pulmonary diseases, heart failure, atrial fibrillation, myocardial infarction, and other venous and arterial thromboembolic conditions [16,22]. RDW levels in patients with those conditions also predict mortality and major adverse events [13,15,16,18,20].

RDW becomes chronically elevated through unknown mechanisms, which could involve inflammation, anemia, or nutritional deficiencies, among other potential pathways [16,18,20]. RDW is, however, a powerful independent predictor of health outcomes in risk prediction models that have adjusted for demographics, cardiometabolic risk factors, C-reactive protein, folate, vitamin B_12_, hemoglobin, iron deficiency, and other factors [13,14,15,16,17,18,19,20]. Chronically elevated RDW may result from tissue-level hypoxemia or another fundamental homeostatic response to disease that stimulates early RBC release from bone marrow, the delay of eryptosis, or both [17,22,23]. As such, RDW is a non-specific indicator of general health status that may indicate the existence of underlying, undiagnosed health problems for people free of chronic diseases and may reveal the severity of disease for people with diagnosed chronic conditions [16]. It may also provide insight into the ability of the human body to respond to external health insults, including infectious pathogens or traumatic harms [14,19] and could be an indicator of frailty. Fortunately, knowing the mechanism by which RDW metrics become elevated is not necessary for using them in guiding health practices.

### 4.3. RDW as an Effect Modifier

Intermittent fasting could be a therapeutic method that modifies health risks marked by elevated RDW. Intermittent fasting reduces weight to a similar extent as caloric restriction in a variety of populations [1,2,3,4] and improves cardiometabolic factors in apparently healthy people and in patients with diagnosed metabolic disease [2,3,4,9]. Little attention in fasting research, though, has been paid to risk related to hematology factors such as RDW. One study examined acute changes in RDW-CV but reported no effect of fasting in changing the level of RDW-CV during the 24-h fast [21]. Because RDW is a powerful predictor of both disease onset and prognosis after disease diagnosis, if intermittent fasting induces long-term changes in RDW, one implication could be that people with elevated RDW may benefit from targeted use of intermittent fasting. In part, this assumes that RDW is in the causal pathway of chronic diseases and that modifying it will reduce the risk of poor health outcomes.

Unfortunately, this study showed that both RDW-SD and RDW-CV were unchanged by the intermittent fasting regimen. These findings may disclose that fasting does not alter RDW. No treatment has been identified that reduces RDW, though, so it is not surprising that no change was observed herein. In contrast, this may have occurred because the low-frequency fasting regimen was insufficient to change RDW metrics, the intervention period of six months may have been too short to observe the changes, or the study population size was too small. Further investigation of the effects of intermittent fasting on RDW measures would help clarify if higher-intensity regimens, longer interventional periods, or larger sample sizes are needed to observe RDW changes.

Because RDW as a biomarker can be used for prognostication of chronic disease development and adverse health events, its level at a single timepoint may identify individuals who could receive more benefit than others if they engage in intermittent fasting. This is likely because people with a higher RDW have a greater need for improved health, especially in terms of insulin resistance. This study suggests that this is the case in which RDW is an effect modifier of intermittent fasting and can be used to guide decision making regarding therapeutic use of fasting and related applications, such as motivating patients to adhere to an intermittent fasting regimen. Given the vast literature on the powerful ability of RDW to predict future health outcomes, measurement of RDW may aid in personalizing the use of intermittent fasting to reduce risk of cardiometabolic disease and events. Potential mechanisms through which intermittent fasting reduces insulin, insulin resistance, and metabolic syndrome include that it causes weight loss [24], it results in the extraction of fatty acids from adipose storage for conversion to ketones to use as energy during cessation of caloric intake [25], and it may result in disposal of energy stores through mitochondrial uncoupling [26], among other mechanisms. Further study of the interaction of RDW together with intermittent fasting is indicated.

### 4.4. Limitations

This was a post hoc analysis of a clinical trial that was not designed to examine RDW-SD or RDW-CV in its primary analyses. Limitations of the trial have been examined previously [9] but include that some factors may have been uncontrolled by the randomization procedure, and findings should be interpreted with caution. The study used a regimen that may have been insufficient to reduce RDW metrics, the sample size was relatively small, and detailed information on dietary intake was not collected, which did not permit examination of differences in nutrition over the course of the study. RDW is connected to nutrition through iron deficiency, which may connect to this study through changes in RDW. Although no intermittent-fasting-related RDW changes were noted, RDW at baseline may have been higher in part due to iron deficiency but iron levels were not measured in this study. Further, the population that was studied consisted of people free of chronic diseases, with a few cardiometabolic risk factors and, thus, may not generalize to other populations. Additional research regarding the findings is required to fully elucidate the relevance of RDW in intermittent fasting. The study did, however, discover a modification of the effect of intermittent fasting in subsets defined by the RDW, and thus the low-frequency fasting regimen did affect certain parameters. Further, subjects in the control group followed their own standard dietary practices that approximate the standard Western diet and reflect real-life dietary behaviors.

## 5. Conclusions

In a post hoc analysis of the WONDERFUL randomized controlled trial, intermittent fasting decreased insulin and other cardiometabolic traits relatively more in subjects whose baseline RDW-SD was elevated (≥median), potentially identifying which patients had greater need of the health intervention. This effect modification on fasting was also found using baseline RDW-CV. These findings suggest that people with greater risk of poor health outcomes, as indicated by RDW level, physiologically respond more to an intermittent fasting therapeutic intervention. RDW may be a readily available, low-cost biomarker for guiding the recommendation of a sustainable intermittent fasting regimen to a patient and a quantitative marker for nudging patients to adhere to intermittent fasting over the long term. Further evaluation of the use of these and other metrics of RBC size dispersion for personalization of intermittent fasting regimens is indicated, including to validate these findings in larger populations over longer intervention periods, to explore the value of using RDW measures to nudge patients to adhere to therapeutic fasting, and to identify people who may be at elevated risk of chronic diseases for whom an intermittent fasting regimen should be initiated because it may provide the largest benefit to them.

## Figures and Tables

**Table 1:** Baseline characteristics of subjects who completed the 26-week study and had a complete blood count laboratory panel, including RDW-SD and RDW-CV, at baseline and 26 weeks.

Characteristic	Overall	RDW-SD < 42.6 fL	RDW-SD ≥ 42.6 fL	*p*-Value
Sample Size	N = 71	*n* = 35	*n* = 36	-----
Randomization				
Intermittent Fasting	53.5%	51.4%	55.6%	0.73
Demographics				
Age (years)	49.1 ± 11.1	46.4 ± 10.2	51.8 ± 11.4	0.042
Age min, max	27.7, 69.9	28.7, 66.7	27.7, 69.9	-----
Sex (female)	64.8%	62.9%	66.7%	0.74
Race (nonwhite)	2.8%	2.9%	2.8%	0.98
Ethnicity (Hispanic)	7.0%	8.6%	5.6%	0.62
Cardiometabolic Risk Factors				
Insulin (mIU/L)	11.2 ± 5.9	10.6 ± 4.7	11.8 ± 6.8	0.40
Glucose (mg/dL)	93.6 ± 12.8	93.3 ± 13.9	93.9 ± 11.8	0.84
HOMA-IR	2.64 ± 1.53	2.46 ± 1.15	2.82 ± 1.83	0.33
Metabolic Syndrome Score	4.05 ± 3.76	3.47 ± 3.96	4.62 ± 3.51	0.20
Weight (kg)	100.7 ± 23.3	95.9 ± 21.2	105.4 ± 24.6	0.09
Body Mass Index (kg/m2)	33.7 ± 7.5	32.3 ± 6.5	35.0 ± 8.3	0.14
Waist Circumference (cm)	105 ± 20	102 ± 19	108 ± 20	0.47
Systolic Blood Pressure (mmHg)	128 ± 12	126 ± 13	131 ± 10	0.06
Diastolic Blood Pressure (mmHg)	81.4 ± 9.1	79.4 ± 10.3	83.4 ± 7.4	0.07
Total Cholesterol (mg/dL)	200 ± 26	203 ± 27	197 ± 26	0.38
LDL Cholesterol (mg/dL)	127 ± 19	130 ± 20	124 ± 18	0.23
HDL Cholesterol (mg/dL)	46.1 ± 11.2	45.3 ± 8.9	46.9 ± 13.2	0.54
Triglycerides (mg/dL)	135 ± 72	141 ± 77	130 ± 67	0.54
Complete Blood Count				
Red Blood Cell Count (×106/μL)	4.88 ± 0.38	4.95 ± 0.43	4.81 ± 0.32	0.11
Hemoglobin (g/dL)	14.5 ± 1.0	14.6 ± 1.1	14.3 ± 0.9	0.22
Hematocrit (%)	43.2 ± 2.7	43.3 ± 2.9	43.1 ± 2.6	0.84
White Blood Cell Count (×103/μL)	6.26 ± 1.58	6.17 ± 1.51	6.36 ± 1.67	0.62
Platelet Count (×106/μL)	249 ± 52	250 ± 53	249 ± 51	0.93
Mean Corpuscular Volume (fL)	88.7 ± 3.8	87.6 ± 3.7	89.8 ± 3.7	0.013
MCH (pg)	30.1 ± 3.9	30.4 ± 5.4	29.8 ± 1.5	0.53
MCHC (g/dL)	33.5 ± 1.1	33.8 ± 1.1	33.2 ± 1.0	0.027
RDW-CV (%)	13.2 ± 0.8	12.8 ± 0.6	13.7 ± 0.8	<0.001
Mean Platelet Volume (fL)	10.2 ± 0.8	10.0 ± 0.7	10.4 ± 0.8	0.02

**Table 2:** Change scores from baseline to 26 weeks for subjects randomized to intermittent fasting (*n* = 38) or control (*n* = 33) groups were stratified by median baseline RDW-SD (<42.6 fL: *n* = 18 fasting, *n* = 17 controls; ≥42.6 fL: *n* = 20 fasting, *n* = 16 controls).

26-Week Change Score	RDW-SD Stratum	Intermittent Fasting	Ad Libitum Control	In-Stratum *p*-Value *	Across-Stratum *p*-Interaction †
Insulin (mIU/L)	<42.6 fL	−1.99 ± 2.80	−1.08 ± 3.40	0.39	-----
	≥42.6 fL	−3.45 ± 2.27	0.48 ± 3.55	<0.001	0.039
Glucose (mg/dL)	<42.6 fL	−5.06 ± 7.47	−4.24 ± 11.23	0.80	-----
	≥42.6 fL	−9.15 ± 10.49	0.13 ± 10.79	0.014	0.08
HOMA-IR	<42.6 fL	−0.52 ± 0.82	−0.28 ± 0.95	0.43	-----
	≥42.6 fL	−0.96 ± 0.73	0.09 ± 1.17	0.002	0.07
Metabolic Syndrome Score	<42.6 fL	−0.45 ± 2.94	0.13 ± 1.74	0.49	-----
	≥42.6 fL	−1.75 ± 2.01	0.49 ± 2.26	0.003	0.13

* This comparison is between the means of the intermittent fasting and ad libitum control groups within each RDW-SD stratum; † this comparison of a statistical interaction evaluates the modification of the effect of intermittent fasting (vs. controls) by high vs. low RDW-SD.

## Data Availability

The data underlying this article cannot be shared publicly due to United States privacy laws. The data will be shared on reasonable request to the corresponding author.
